# Supplementary material for: Experimental genital tract infection demonstrates Neisseria gonorrhoeae MtrCDE efflux pump is not required for in vivo human infection and identifies gonococcal colonization bottleneck
Source: PLoS Pathog. 2024 Sep 25;20(9):e1012578. doi: 10.1371/journal.ppat.1012578 (PMC11457995; doi:10.1371/journal.ppat.1012578)
Supplement: S1 Fig — (DOCX) [file ppat.1012578.s001.docx]

**S1 Fig**. Duplex Taqman real-time PCR assay design.

Schematic of the duplex Taqman real-time PCR assay design used to distinguish between wild type and mutant FA1090 and FA19 strains is shown in panel A and theoretical detection scenarios are shown in panel B. In both panels, the wild-type specific probe (5’FAM labeled), amplicon, and FAM-based detection are shown in pink. The mutant specific probe (5’HEX), amplicon and HEX-based detection are shown in teal. The *mtrD* gene in FA1090 and FA19 share 100% genetic homology.

A. The wild-type specific primers and probe were purposefully designed to be located within the *mtrD* gene, which is deleted in the mutant strains; the resulting amplicon is a 164bp product that emits FAM fluorescence. The primers and probe targeting the mutant strain were designed in a gap PCR fashion, whereby the forward and reverse primers flank the *mtrD* deletion and the probe spans either side of the deletion; only if a deletion is present, can the real-time assay make use of these primers and probe to amplify and detect the 221 bp product, which emits HEX fluorescence with each round of amplification. In the absence of the gene deletion (i.e., if a wild-type strain were present), the resulting product would be approximately 3,000 base pirs long, whose amplification efficiency with real-time chemistry is virtually nil, and thus no HEX-based fluorescence is detected.

B Only wells with detection signal for either FAM (wild-type specific amplification) or HEX (mutant specific amplification), but not both, were evaluable and were included in strain determination analyses. Wells in which both fluorophores were detected or had no detectable/amplified PCR product were deemed not evaluable and were not included in strain determination analyses.
