# Supplementary material for: Experimental genital tract infection demonstrates Neisseria gonorrhoeae MtrCDE efflux pump is not required for in vivo human infection and identifies gonococcal colonization bottleneck
Source: PLoS Pathog. 2024 Sep 25;20(9):e1012578. doi: 10.1371/journal.ppat.1012578 (PMC11457995; doi:10.1371/journal.ppat.1012578)
Supplement: S1 Table — (DOCX) [file ppat.1012578.s002.docx]

**S1 Table.** Real-time PCR primers, probes, and reaction recipes

| **Component** | **Sequence** | **Working concentration (mM)** | **Volume per reaction (μL)** | **Final concentration per reaction (mM)** |
| --- | --- | --- | --- | --- |
| Bio-Rad iQ Multiplex Powermix | N/A | 2x | 6 | 1x |
| Forward primer specific to wild-type strain | TCGTCTTATGTCAGCGACTTC | 10mM | 0.6 | 0.5 |
| Reverse primer specific to wild-type strain | TCCCAAGAAACAGTAGCAATG | 10mM | 0.6 | 0.5 |
| Forward primer specific to *mtrD* deleted mutant strain | CGAAAAAGGTAACGCCTAAAG | 10mM | 0.6 | 0.5 |
| Reverse primer specific to *mtrD* deleted mutant strain | CTGCAACAGAGGTCAAGGTAG | 10mM | 0.6 | 0.5 |
| Taqman probe specific to wild-type strain | 5’FAM-GTATGCAGCCTGCCGATATT-3’BHQ-1 | 10mM | 0.25 | 0.2 |
| Taqman probe specific to *mtrD* deleted mutant strain | 5’HEX-AAGCCAAACCTGCTTCTGAA-3’BHQ-1 | 10mM | 0.25 | 0.2 |
| Water | N/A | - | 1.1 | - |
