# Supplementary material for: Experimental genital tract infection demonstrates Neisseria gonorrhoeae MtrCDE efflux pump is not required for in vivo human infection and identifies gonococcal colonization bottleneck
Source: PLoS Pathog. 2024 Sep 25;20(9):e1012578. doi: 10.1371/journal.ppat.1012578 (PMC11457995; doi:10.1371/journal.ppat.1012578)
Supplement: S2 Table — (DOCX) [file ppat.1012578.s003.docx]

**S2 Table.** Strain composition of the gonococcal population in mixed inocula used in the four inoculation cohorts of the human challenge studies and of gonococci recovered from first void urine of men from treatment day when clinical urethritis was apparent. The participants shown in grey (280 and 282) were not evaluable and their data were not included in final analyses.

| **Cohort** | **Participant ID^a^** | **Infected?** | **Included in final analyses** | **Day post-inoculation** | **Treatment day post-inoculation** | **% (n^b^) FA1090∆*mtrD* recovered on treatment day** | **% (n^b^) FA1090 recovered on treatment day** | **Mutant cfu recovered from urine / wild-type cfu recovered from urine on treatment day (A)^c^** | **% (n^b^) FA1090∆*mtrD* in inoculum** | **% (n^b^) FA1090 wild-type in inoculum** | **Number of mutant cfu inoculum / number of wild-type cfu inoculum on treatment day (B)^c^** | **Competitive Index (CI) A/B** | **Log_10_(CI)** |
| --- | --- | --- | --- | --- | --- | --- | --- | --- | --- | --- | --- | --- | --- |
| 1 | *280^d^* | *Not evaluable* | *No* | *NA* | *NA* | *NA* | *NA* | *NA* | 58.0 (56) | 42.0 (40) | 1.4 | *NA* | *NA* |
|  | *282^e^* | *Not evaluable* | *No* | *NA* | *NA* | *NA* | *NA* | *NA* |  |  |  | *NA* | *NA* |
|  | 281 | Yes | Yes | 1 | 2 | 0.0 (0) | 100 (96) | 0.01 |  |  |  | 0.01 | -2.13 |
|  |  |  |  | 2 |  | 0.0 (0) | 100 (96) | 0.01 |  |  |  | 0.01 | -2.13 |
| 2 | 291 | Yes | Yes | 1 | 4 | 94.0 (82) | 4.7 (4) | 20.50 | 54.9 (45) | 45.1 (37) | 1.22 | 16.86 | 1.23 |
|  |  |  |  | 2 |  | 99.0 (90) | 1 (1) | 90.00 |  |  |  | 74.00 | 1.87 |
|  |  |  |  | 3 |  | 100.0 (93) | 0.0 (0) | 93.00 |  |  |  | 76.47 | 1.88 |
|  |  |  |  | 4 |  | 100.0 (96) | 0.0 (0) | 96.00 |  |  |  | 78.93 | 1.90 |
|  | 292 | Yes | Yes | 1 | 4 | 100.0 (10) | 0.0 (0) | 10.00 |  |  |  | 8.20 | 0.91 |
|  |  |  |  | 2 |  | 100.0 (50) | 0.0 (0) | 50.00 |  |  |  | 41.11 | 1.61 |
|  |  |  |  | 3 |  | 100.0 (70) | 0.0 (0) | 70.00 |  |  |  | 57.56 | 1.76 |
|  |  |  |  | 4 |  | 100.0 (75) | 0.0 (0.0) | 75.00 |  |  |  | 61.67 | 1.79 |
| 3 | 300 | Yes | Yes | 1 | 4 | 0.0 (0) | 100.0 (92) | 0.01 | 55.4 (97) | 44.6 (78) | 1.24 | 0.01 | -2.06 |
|  |  |  |  | 2 |  | 1.0 (1) | 99.0 (95) | 0.01 |  |  |  | 0.01 | -2.07 |
|  |  |  |  | 3 |  | 1.0 (1) | 99.0 (79) | 0.01 |  |  |  | 0.01 | -1.99 |
|  |  |  |  | 4 |  | 0.0 (0) | 100.0 (93) | 0.01 |  |  |  | 0.01 | -2.06 |
|  | 301 | Yes | Yes | 1 | 3 | 0.0 (0) | 0.0 (0) | NA |  |  |  | NA | NA |
|  |  |  |  | 2 |  | 0.0 (0) | 100.0 (96) | 0.01 |  |  |  | 0.01 | -2.08 |
|  |  |  |  | 3 |  | 0.0 (0) | 100.0 (87) | 0.01 |  |  |  | 0.01 | -2.03 |
|  | 302 | Yes | Yes | 1 | 2 | 71.6 (53) | 28.4 (21) | 2.52 |  |  |  | 2.03 | 0.31 |
|  |  |  |  | 2 |  | 8.9 (7) | 91.1 (72) | 0.10 |  |  |  | 0.08 | -1.11 |
|  | 303 | Yes | Yes | 1 | 4 | 100.0 (14) | 0.0 (0) | 14.00 |  |  |  | 11.26 | 1.05 |
|  |  |  |  | 2 |  | 0.0 (0) | 0.0 (0) | NA |  |  |  | NA | NA |
|  |  |  |  | 3 |  | 0.0 (0) | 0.0 (0) | NA |  |  |  | NA | NA |
|  |  |  |  | 4 |  | 100.0 (90) | 0.0 (0) | 90.00 |  |  |  | 72.37 | 1.86 |
| 4 | 311 | Yes | Yes | 1 | 2 | 0.0 (0) | 100.0 (10) | 0.10 | 75.5 (71) | 24.5 (23) | 3.09 | 0.03 | -1.49 |
|  |  |  |  | 2 |  | 0.0 (0) | 100.0 (40) | 0.03 |  |  |  | 0.01 | -2.09 |
|  | 313 | Yes | Yes | 1 | 4 | 6.3 (4) | 93.7 (59) | 0.07 |  |  |  | 0.02 | -1.66 |
|  |  |  |  | 2 |  | 95.6 (43) | 4.4 (2) | 21.50 |  |  |  | 6.96 | 0.84 |
|  |  |  |  | 3 |  | 100.0 (90) | 0.0 (0) | 90.00 |  |  |  | 29.15 | 1.46 |
|  |  |  |  | 4 |  | 100.0 (96) | 0.0 (0) | 96.00 |  |  |  | 31.10 | 1.49 |
|  | 314 | Yes | Yes | 1 | 4 | 0.0 (0) | 0.0 (0) | NA |  |  |  | NA | NA |
|  |  |  |  | 2 |  | 100.0 (34) | 0.0 (0) | 34.00 |  |  |  | 11.01 | 1.04 |
|  |  |  |  | 3 |  | 100.0 (79) | 0.0 (0) | 79.00 |  |  |  | 25.57 | 1.41 |
|  |  |  |  | 4 |  | 100.0 (93) | 0.0 (0) | 93.00 |  |  |  | 30.13 | 1.48 |

^a^All inoculated participants. Urine culture negative participants at day 5 after inoculation are shown in grey italics.

^b^Number of colony forming units (cfu)

^c^For competitive infections, the culture limit of detection was assigned as 1 cfu/total number of recovered cfu for that sample. This means for the calculation of competitive indices, values of zero recovered cfu were transformed to 1 recovered cfu.

^d^This participant was urine culture-negative throughout the study

^e^This participant did not comply with the first void urine requirement for bacterial cultures.
