# Supplementary material for: Experimental genital tract infection demonstrates Neisseria gonorrhoeae MtrCDE efflux pump is not required for in vivo human infection and identifies gonococcal colonization bottleneck
Source: PLoS Pathog. 2024 Sep 25;20(9):e1012578. doi: 10.1371/journal.ppat.1012578 (PMC11457995; doi:10.1371/journal.ppat.1012578)
Supplement: S2 Fig — (DOCX) [file ppat.1012578.s004.docx]

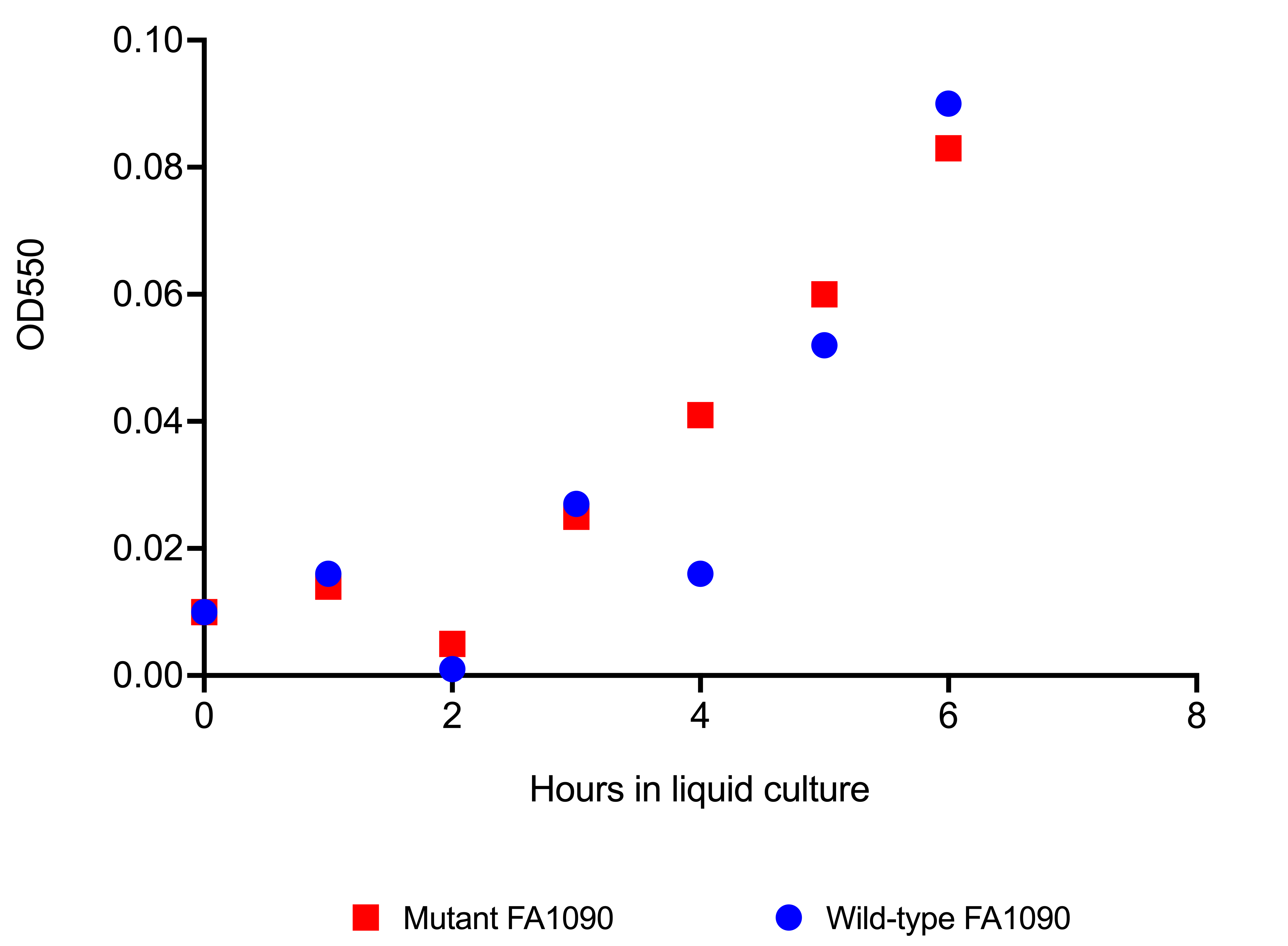


**S2 Fig**. *In vitro* growth curve of FA1090 and FA1090∆*mtrD* isogenic mutant when grown side-by-side in liquid GCB broth with supplements, as evidenced by optical density measurements (OD_550_).
