# Supplementary material for: Experimental genital tract infection demonstrates Neisseria gonorrhoeae MtrCDE efflux pump is not required for in vivo human infection and identifies gonococcal colonization bottleneck
Source: PLoS Pathog. 2024 Sep 25;20(9):e1012578. doi: 10.1371/journal.ppat.1012578 (PMC11457995; doi:10.1371/journal.ppat.1012578)
Supplement: S3 Table — (DOCX) [file ppat.1012578.s005.docx]

**S3 Table.** Strain composition by colony real-time PCR and competitive indices on final culture positive day in mouse challenge studies with wild-type FA1090 and FA1090Δ*mtrD.* Strain composition was determined by colony real-time PCR for the mixed inocula used in these experiments and for gonococci recovered from mouse genital swabs collected on final culture positive day. The final culture positive day was chosen as the end timepoint for fitness analyses because by this time post-inoculation recvered gonococci were deemed to be the ones that successfully established and maintain colonization in the murine genital tract, which is an established approach for analyzing results of competitive gonococcal infections in this mouse model. The genital tract of mice was sampled on days 1, 3, 5, and 7 post-inoculation. All mice were culture negative by day 7. For a more detailed view of longitudinal dynamics of strain compositions related to these experiments please refer to S4 Table.

| **Cohort** | **Mouse** | **% (n cfu) mutant cfu in inoculum** | **% (n cfu) wild-type cfu in inoculum** | **Infected?** | **% (n cfu) mutant cfu recovered on final day** | **% (n cfu) wild-type cfu recovered on final day** | **Number of mutant cfu inoculum / number of wild-type cfu inoculum (A)** | **Mutant cfu recovered from mouse / wild-type cfu recovered from mouse (B)** | **Competitive Index (CI) (B/A)** | **Log_10_(CI)** |
| --- | --- | --- | --- | --- | --- | --- | --- | --- | --- | --- |
| 1 | 1 | 60.0 (45) | 40.0 (30) | Y | 0.0 (0) | 100.0 (90) | 1.5 | 0.01 | 0.01 | -2.13 |
|  | 2 |  |  | Y | 0.0 (0) | 100.0 (91) |  | 0.01 | 0.01 | -2.14 |
|  | 3 |  |  | Y | 80.2 (69) | 19.8 (17) |  | 4.06 | 2.71 | 0.43 |
|  | 4 |  |  | Y | 81.9 (77) | 18.1 (17) |  | 4.53 | 3.02 | 0.48 |
| 2 | 5 | 39.6 (19) | 60.4 (29) | Y | 100.0 (35) | 0.0 (0) | 0.66 | 35.00 | 53.42 | 1.73 |
|  | 6 |  |  | Y | 97.6 (41) | 2.4 (1) |  | 41.00 | 62.58 | 1.80 |
|  | 7 |  |  | Y | 0.0 (0) | 100.0 (40) |  | 0.03 | 0.04 | -1.42 |
|  | 8 |  |  | Y | 93.2 (41) | 6.8 (3) |  | 13.67 | 20.86 | 1.32 |
|  | 9 |  |  | Y | 30.8 (12) | 69.2 (27) |  | 0.44 | 0.68 | -0.17 |
|  | 10 |  |  | Y | 6.7 (3) | 93.3 (42) |  | 0.07 | 0.11 | -0.96 |
|  | 11 |  |  | Y | 100.0 (45) | 0.0 (0) |  | 45.00 | 68.68 | 1.84 |
| Average inoculum | | 49.8 | 50.2 | NA | NA | NA | NA | NA | NA | NA |
