# Supplementary material for: Experimental genital tract infection demonstrates Neisseria gonorrhoeae MtrCDE efflux pump is not required for in vivo human infection and identifies gonococcal colonization bottleneck
Source: PLoS Pathog. 2024 Sep 25;20(9):e1012578. doi: 10.1371/journal.ppat.1012578 (PMC11457995; doi:10.1371/journal.ppat.1012578)
Supplement: S4 Table — (DOCX) [file ppat.1012578.s006.docx]

**S4 Table.** Strain composition by colony real-time PCR and competitive indices in mouse challenge studies with mixtures of wild-type FA1090 and FA1090Δ*mtrD* and mixtures containing FA19 and FA19Δ*mtrD.* Strain composition was determined by colony real-time PCR for the mixed inocula used in these experiments and for gonococci recovered from mouse genital swabs collected from each positive culture day. The genital tract of mice was sampled on days 1, 3, 5, and 7 post-inoculation. For cohort 1 of mice inoculated with FA1090 + FA1090Δ*mtrD*, only single colonies from last day of positive cultures were included in the colony real-time PCR analyses. For subsequent experiments, colony real-time PCR was performed on single colony isolates from all culture positive days. All mice were culture negative by day 7, hence no day 7 data are available.

| **Mouse** | **Cohort** | **Strains in inoculum** | **Number of mutant CFU in inoculum** | **Number of wild-type CFU in inoculum** | **Mutant CFU inoculum / wild-type CFU inoculum (A)** | **Day of sampling post-challenge** | **Last day of positive cultures** | **Number of colonies available for screening by qPCR** | **Number of colonies evaluable for qPCR results** | **Number mutant CFU recovered from mouse vaginal swab on final day** | **Number wild-type CFU recovered from mouse vaginal swab on final day** | **Number mutant CFU recovered on final day (transformed)** | **Number wild-type CFU recovered on final day (transformed)** | **Mutant CFU output from mouse / wild-type CFU output from mouse (B)** | **Competitive Index (CI) (B/A)** | **Log10(CI)** |
| --- | --- | --- | --- | --- | --- | --- | --- | --- | --- | --- | --- | --- | --- | --- | --- | --- |
| 1 | 1 | FA1090 + FA1090 Δ*mtrD* | 45 | 30 | 1.50 | day5 | day5 | 96 | 90 | 0 | 90 | 1 | 90 | 0.01 | 0.01 | -2.13 |
| 2 |  |  |  |  |  | day5 | day5 | 96 | 91 | 0 | 91 | 1 | 91 | 0.01 | 0.01 | -2.14 |
| 3 |  |  |  |  |  | day1 | day1 | 91 | 86 | 69 | 17 | 69 | 17 | 4.06 | 2.71 | 0.43 |
| 4 |  |  |  |  |  | day3 | day3 | 96 | 94 | 77 | 17 | 77 | 17 | 4.53 | 3.02 | 0.48 |
| 5 | 2 | FA1090 + FA1090 Δ*mtrD* | 19 | 29 | 0.66 | day1 | day3 | 48 | 42 | 24 | 12 | 24 | 12 | 2.00 | 3.05 | 0.48 |
| 5 |  |  |  |  |  | day3 | day3 | 48 | 35 | 35 | 0 | 35 | 1 | 35.00 | 53.42 | 1.73 |
| 6 |  |  |  |  |  | day1 | day3 | 48 | 48 | 48 | 0 | 48 | 1 | 48.00 | 73.26 | 1.86 |
| 6 |  |  |  |  |  | day3 | day3 | 48 | 42 | 41 | 1 | 41 | 1 | 41.00 | 62.58 | 1.80 |
| 7 |  |  |  |  |  | day 1 | day5 | 48 | 45 | 12 | 33 | 12 | 33 | 0.36 | 0.56 | -0.26 |
| 7 |  |  |  |  |  | day3 | day5 | 48 | 47 | 16 | 31 | 16 | 31 | 0.52 | 0.79 | -0.10 |
| 7 |  |  |  |  |  | day5 | day5 | 48 | 40 | 0 | 40 | 1 | 40 | 0.03 | 0.04 | -1.42 |
| 8 |  |  |  |  |  | day1 | day5 | 48 | 48 | 37 | 11 | 37 | 11 | 3.36 | 5.13 | 0.71 |
| 8 |  |  |  |  |  | day3 | day5 | 48 | 48 | 35 | 13 | 35 | 13 | 2.69 | 4.11 | 0.61 |
| 8 |  |  |  |  |  | day5 | day5 | 48 | 44 | 41 | 3 | 41 | 3 | 13.67 | 20.86 | 1.32 |
| 9 |  |  |  |  |  | day1 | day5 | 48 | 48 | 17 | 31 | 17 | 31 | 0.55 | 0.84 | -0.08 |
| 9 |  |  |  |  |  | day3 | day5 | 48 | 48 | 3 | 45 | 3 | 45 | 0.07 | 0.10 | -0.99 |
| 9 |  |  |  |  |  | day5 | day5 | 48 | 39 | 12 | 27 | 12 | 27 | 0.44 | 0.68 | -0.17 |
| 10 |  |  |  |  |  | day1 | day5 | 48 | 48 | 0 | 48 | 1 | 48 | 0.02 | 0.03 | -1.50 |
| 10 |  |  |  |  |  | day3 | day5 | 48 | 31 | 1 | 30 | 1 | 30 | 0.03 | 0.05 | -1.29 |
| 10 |  |  |  |  |  | day5 | day5 | 48 | 45 | 3 | 42 | 3 | 42 | 0.07 | 0.11 | -0.96 |
| 11 |  |  |  |  |  | day1 | day5 | 48 | 48 | 48 | 0 | 48 | 1 | 48.00 | 73.26 | 1.86 |
| 11 |  |  |  |  |  | day3 | day5 | 48 | 48 | 48 | 0 | 48 | 1 | 48.00 | 73.26 | 1.86 |
| 11 |  |  |  |  |  | day5 | day5 | 48 | 45 | 45 | 0 | 45 | 1 | 45.00 | 68.68 | 1.84 |
| 12 | 3 | FA19 + FA19 Δ*mtrD* | 28 | 20 | 1.40 | day1 | day5 | 48 | 36 | 0 | 36 | 1 | 36 | 0.03 | 0.02 | -1.70 |
| 12 |  |  |  |  |  | day3 | day5 | 48 | 22 | 0 | 22 | 1 | 22 | 0.05 | 0.03 | -1.49 |
| 12 |  |  |  |  |  | day5 | day5 | 48 | 44 | 0 | 44 | 1 | 44 | 0.02 | 0.02 | -1.79 |
| 13 |  |  |  |  |  | day1 | day5 | 48 | 35 | 0 | 35 | 1 | 35 | 0.03 | 0.02 | -1.69 |
| 13 |  |  |  |  |  | day3 | day5 | 48 | 16 | 0 | 16 | 1 | 16 | 0.06 | 0.04 | -1.35 |
| 13 |  |  |  |  |  | day5 | day5 | 48 | 47 | 2 | 45 | 2 | 45 | 0.04 | 0.03 | -1.50 |
| 14 |  |  |  |  |  | day1 | day5 | 48 | 48 | 5 | 43 | 5 | 43 | 0.12 | 0.08 | -1.08 |
| 14 |  |  |  |  |  | day3 | day5 | 48 | 46 | 5 | 41 | 5 | 41 | 0.12 | 0.09 | -1.06 |
| 14 |  |  |  |  |  | day5 | day5 | 48 | 23 | 0 | 23 | 1 | 23 | 0.04 | 0.03 | -1.51 |
| 15 |  |  |  |  |  | day1 | day3 | 48 | 45 | 11 | 34 | 11 | 34 | 0.32 | 0.23 | -0.64 |
| 15 |  |  |  |  |  | day3 | day3 | 48 | 39 | 1 | 38 | 1 | 38 | 0.03 | 0.02 | -1.73 |
| 16 |  |  |  |  |  | day1 | day3 | 48 | 48 | 0 | 48 | 1 | 48 | 0.02 | 0.01 | -1.83 |
| 16 |  |  |  |  |  | day3 | day3 | 48 | 37 | 0 | 37 | 1 | 37 | 0.03 | 0.02 | -1.71 |
| 17 |  |  |  |  |  | day1 | day1 | 48 | 38 | 2 | 36 | 2 | 36 | 0.06 | 0.04 | -1.40 |
| 18 |  |  |  |  |  | day1 | day5 | 48 | 41 | 33 | 8 | 33 | 8 | 4.13 | 2.95 | 0.47 |
| 18 |  |  |  |  |  | day3 | day5 | 48 | 36 | 30 | 6 | 30 | 6 | 5.00 | 3.57 | 0.55 |
| 18 |  |  |  |  |  | day5 | day5 | 48 | 39 | 38 | 1 | 38 | 1 | 38.00 | 27.14 | 1.43 |
