# Supplementary material for: Experimental genital tract infection demonstrates Neisseria gonorrhoeae MtrCDE efflux pump is not required for in vivo human infection and identifies gonococcal colonization bottleneck
Source: PLoS Pathog. 2024 Sep 25;20(9):e1012578. doi: 10.1371/journal.ppat.1012578 (PMC11457995; doi:10.1371/journal.ppat.1012578)
Supplement: S5 Table — (DOCX) [file ppat.1012578.s007.docx]

**S5 Table**. ***In vitro* competitive growth culture of FA1090 and FA1090∆*mtrD****.* A liquid culture competitive growth experiment was conducted using bacteria from the same stock of FA1090 and FA1090Δ*mtrD* mixture as used in the human and mouse challenge studies. This experiment was conducted in triplicate (i.e. using three individual, independent vials of bacteria from the same working bank lot of FA1090 and FA1090Δ*mtrD* mixtures as used in human and mouse competitive infections). The procedure was as follows: three vials of FA1090 +FA1090Δ*mtrD* mixtures were thawed, and bacteria were sub-cultured onto five individual GCB agar plates for each vial (15 plates total). The next day, three inoculum suspensions were made using the same procedures as for *in vivo* challenge studies. The three replicate bacterial suspensions in 6mL sterile PBS each with the same OD_550_ of 0.3 were placed in liquid culture (34mL of GCB with supplements). Every hour up to 4 hours, 4 mL aliquots of liquid culture were removed and centrifuged to obtain culture sediments. DNA from culture sediments was extracted. The DNA of the 0h and 4h timepoints were whole genome sequenced on the Illumina platform (2x150bp). Paired quality filtered reads were iteratively mapped with 5 iterations to FA1090 reference sequence (Genbank accession number [AE004969.1](https://www.ncbi.nlm.nih.gov/nuccore/AE004969.1)). All reads were mapped in parallel to the AE004969.1 FA1090 reference sequences (wild-type reference sequence for making wild-type read calls) and to another reference sequence derived from AE004969.1 FA1090 from which we removed the *mtrD* gene sequence (mutant reference sequence for making mutant read calls). We allowed for 75bp 5’ and 3’ flanking sequences for mapping of both wild-type and mutant reads to increase accuracy of strain calling and enumeration. The deep sequencing data show that there is no difference in *in vitro* competitive fitness between the two strains at the *mtrD* locus after 4 hours of competitive growth in liquid broth.

| **Vial ID** | **Number of mutant reads at 0h** | **Number of wild-type reads at 0h** | **Mutant reads/wild-type reads at 0h** | **Number of mutant reads at 4h** | **Number of wild-type reads at 4h** | **Mutant reads/wild-type reads at 4h** | **Competitive Index (CI) (Column D/Column G)** | **Log10 (CI)** |
| --- | --- | --- | --- | --- | --- | --- | --- | --- |
| vial 1 | 2514 | 2340 | 0.93 | 2648 | 2418 | 1.10 | 0.85 | -0.07 |
| vial 2 | 2456 | 2654 | 1.08 | 2648 | 2511 | 1.05 | 1.02 | 0.01 |
| vial 3 | 2267 | 2413 | 1.06 | 1864 | 2012 | 0.93 | 1.15 | 0.06 |
