# Supplementary material for: Experimental genital tract infection demonstrates Neisseria gonorrhoeae MtrCDE efflux pump is not required for in vivo human infection and identifies gonococcal colonization bottleneck
Source: PLoS Pathog. 2024 Sep 25;20(9):e1012578. doi: 10.1371/journal.ppat.1012578 (PMC11457995; doi:10.1371/journal.ppat.1012578)
Supplement: S6 Table — (DOCX) [file ppat.1012578.s008.docx]

**S6 Table.** Strain composition by quantitative culture in mixed inocula used in mouse challenge studies with wild-type FA19 and FA19*mtrD*::Kan and of gonococci recovered from mouse genital swabs collected from each positive culture day. The FA19*mtrD*::Kan strain is resistant to kanamycin. The number of strain-specific cfu in the 2 inocula used for the two groups of mice and among mouse genital isolates was determined as follows: we cultured equal volumes of diluted inoculum and vaginal swab suspensions on GC agar containing streptomycin (to recover and enumerate total gonococci) and GC agar containing streptomycin and kanamycin (to recover and enumerate mutant gonococci). We divided the number of kanamycin resistant cfu by the total number of gonococci recovered. For all mouse infection experiments, the limit of detection was 1 cfu per 100μl of vaginal swab suspension.

| **Mouse** | **Inoculation group/cohort** | **Strains in inoculum** | **Number of mutant cfu in inoculum** | **Number of wild-type cfu in inoculum** | **Mutant cfu inoculum / wild-type cfu inoculum (A)** | **Day of sampling post-challenge** | **Last day of positive cultures** | **Number mutant cfu recovered from mouse genital swab** | **Number wild-type cfu recovered from mouse genital swab** | **Mutant cfu from mouse / wild-type cfu from mouse (B)** | **Competitive index (CI) (B/A)** | **Log 10 (CI)** |
| --- | --- | --- | --- | --- | --- | --- | --- | --- | --- | --- | --- | --- |
| Mouse 1 | 1 | FA19*mtrD::Kan + FA19* | 69000000 | 58000000 | 1.189655172 | day 1 | day 5 | 1 | 1596320 | 6.26441E-07 | 5.26573E-07 | -6.27854105 |
| Mouse 1 |  |  | 69000000 | 58000000 | 1.189655172 | day 3 | day 5 | 1 | 1079999 | 9.25927E-07 | 7.78315E-07 | -6.10884445 |
| Mouse 1 |  |  | 69000000 | 58000000 | 1.189655172 | day 5 | day 5 | 1 | 167999 | 5.95E-06 | 5.00348E-06 | -5.30072779 |
| Mouse 2 |  |  | 69000000 | 58000000 | 1.189655172 | day 1 | day 5 | 1 | 1907200 | 5.24329E-07 | 4.4074E-07 | -6.35581734 |
| Mouse 2 |  |  | 69000000 | 58000000 | 1.189655172 | day 5 | day 5 | 1 | 167999 | 5.95E-06 | 5.00348E-06 | -5.30072779 |
| Mouse 2 |  |  | 69000000 | 58000000 | 1.189655172 | day 3 | day 5 | 1 | 1279999 | 7.81251E-07 | 6.56703E-07 | -6.18263073 |
| Mouse 3 |  |  | 69000000 | 58000000 | 1.189655172 | day 1 | day 5 | 1 | 1800000 | 5.55556E-07 | 4.66989E-07 | -6.3306936 |
| Mouse 3 |  |  | 69000000 | 58000000 | 1.189655172 | day 3 | day 5 | 1 | 75999 | 1.31581E-05 | 1.10604E-05 | -4.95622898 |
| Mouse 3 |  |  | 69000000 | 58000000 | 1.189655172 | day 5 | day 5 | 1 | 215999 | 4.62965E-06 | 3.89159E-06 | -5.40987284 |
| Mouse 4 |  |  | 69000000 | 58000000 | 1.189655172 | day 1 | day 3 | 1 | 317200 | 3.15259E-06 | 2.65E-06 | -5.57675428 |
| Mouse 4 |  |  | 69000000 | 58000000 | 1.189655172 | day 3 | day 3 | 1 | 239999 | 4.16668E-06 | 3.50243E-06 | -5.45563053 |
| Mouse 4 |  |  | 69000000 | 58000000 | 1.189655172 | day 5 | day 3 | negative | negative |  |  |  |
| Mouse 5 |  |  | 69000000 | 58000000 | 1.189655172 | day 1 | day 1 | 1 | 29899 | 3.34459E-05 | 2.8114E-05 | -4.55107776 |
| Mouse 5 |  |  | 69000000 | 58000000 | 1.189655172 | day 3 | day 1 | negative | negative |  |  |  |
| Mouse 5 |  |  | 69000000 | 58000000 | 1.189655172 | day 5 | day 1 | negative | negative |  |  |  |
| Mouse 6 |  |  | 69000000 | 58000000 | 1.189655172 | day 1 | day 5 | 440 | 239560 | 0.001836701 | 0.001543893 | -2.81138273 |
| Mouse 6 |  |  | 69000000 | 58000000 | 1.189655172 | day 3 | day 5 | 1 | 1049 | 0.000953289 | 0.000801315 | -3.09619659 |
| Mouse 6 |  |  | 69000000 | 58000000 | 1.189655172 | day 5 | day 5 | 1 | 639 | 0.001564945 | 0.001315461 | -2.88092196 |
| Mouse 10 | 2 | FA19*mtrD::Kan + FA19* | 89000000 | 73000000 | 1.219178082 | day 1 | day 3 | 1 | 1 | 0 | 0 |  |
| Mouse 10 |  |  | 89000000 | 73000000 | 1.219178082 | day 3 | day 3 | 1 | 35 | 0.028571429 | 0.023434992 | -1.63013519 |
| Mouse 10 |  |  | 89000000 | 73000000 | 1.219178082 | day 5 | day 3 | negative | negative |  |  |  |
| Mouse 11 |  |  | 89000000 | 73000000 | 1.219178082 | day 1 | day 5 | 1 | 30399 | 3.28958E-05 | 2.6982E-05 | -4.56892644 |
| Mouse 11 |  |  | 89000000 | 73000000 | 1.219178082 | day 3 | day 5 | 1 | 22399 | 4.46449E-05 | 3.66188E-05 | -4.43629578 |
| Mouse 11 |  |  | 89000000 | 73000000 | 1.219178082 | day 5 | day 5 | 1 | 115999 | 8.62076E-06 | 7.07096E-06 | -5.15052139 |
| Mouse 12 |  |  | 89000000 | 73000000 | 1.219178082 | day 1 | day 5 | 6000000 | 320000 | 18.75 | 15.37921348 | 1.186934126 |
| Mouse 12 |  |  | 89000000 | 73000000 | 1.219178082 | day 3 | day 5 | 1 | 18399 | 5.43508E-05 | 4.45799E-05 | -4.35086137 |
| Mouse 12 |  |  | 89000000 | 73000000 | 1.219178082 | day 5 | day 5 | 1 | 10799 | 9.26012E-05 | 7.59538E-05 | -4.11945069 |
| Mouse 7 |  |  | 89000000 | 73000000 | 1.219178082 | day 1 | day 1 | 1 | 300000 | 3.33333E-06 | 2.73408E-06 | -5.5631884 |
| Mouse 7 |  |  | 89000000 | 73000000 | 1.219178082 | day 3 | day 1 | negative | negative |  |  |  |
| Mouse 7 |  |  | 89000000 | 73000000 | 1.219178082 | day 5 | day 1 | negative | negative |  |  |  |
| Mouse 8 |  |  | 89000000 | 73000000 | 1.219178082 | day 1 | day 1 | 1 | 239 | 0.0041841 | 0.003431903 | -2.46446505 |
| Mouse 8 |  |  | 89000000 | 73000000 | 1.219178082 | day 3 | day 1 | negative | negative |  |  |  |
| Mouse 8 |  |  | 89000000 | 73000000 | 1.219178082 | day 5 | day 1 | negative | negative |  |  |  |
| Mouse 9 |  |  | 89000000 | 73000000 | 1.219178082 | day 1 | day 5 | 2800 | 161200 | 0.017369727 | 0.014247079 | -1.84627415 |
| Mouse 9 |  |  | 89000000 | 73000000 | 1.219178082 | day 3 | day 5 | 1 | 51999 | 1.92311E-05 | 1.57739E-05 | -4.80206214 |
| Mouse 9 |  |  | 89000000 | 73000000 | 1.219178082 | day 5 | day 5 | 1 | 3999999 | 2.5E-07 | 2.05056E-07 | -6.68812703 |
