# Supplementary material for: Experimental genital tract infection demonstrates Neisseria gonorrhoeae MtrCDE efflux pump is not required for in vivo human infection and identifies gonococcal colonization bottleneck
Source: PLoS Pathog. 2024 Sep 25;20(9):e1012578. doi: 10.1371/journal.ppat.1012578 (PMC11457995; doi:10.1371/journal.ppat.1012578)
Supplement: Attachment — Submitted filename: Waltmann-Response-1Sept2024-Final.docx [file ppat.1012578.s009.docx]

Experimental genital tract infection demonstrates *Neisseria gonorrhoeae* MtrCDE efflux pump is not required for *in vivo* human infection and identifies gonococcal colonization bottleneck

Responses to Reviewer's Questions

Dear reviewers and editor,

We are grateful to the reviewers for the careful review of our manuscript. Our manuscript has benefitted from their suggestions and is a much stronger piece of work now. Please find our responses below in blue. The line numbers to those in the unmarked (clean) revised version of the manuscript.

Sincerely,
Andreea Waltmann

Reviewer #1: In this manuscript, Waltmann et al. examines the role of the MtrCDE efflux pump of Neisseria gonorrhoeae in human and murine models of experimental infection. They show that the efflux pump is not required for infection of men in a strain that is not a strong expressor of the pump, provide evidence that a bottleneck exists during infection of the male urethra, and show strain differences (in an expressor vs. an under-expressor) in the requirement for expression of MtrCDE in female mice. As such, the data is novel and important, especially the evidence for a bottleneck during infection of male volunteers.

We thank the reviewer for their positive feedback on our manuscript.

Reviewer #2: The initial aim of this manuscript is to assess the potential in vivo role of the MtrCDE efflux pump using the human challenge model. Using FA1090 the authors were not able to show a difference between the WT and mutant strain and concluded that in this model the efflux pump was not responsible for a competitive advantage. On the other hand, from their data they identified a bottleneck for GC infection. Furthermore as these results were apparently contradictory to previously published data showing a role of this locus using another strain, the authors tested their strain in a mice model and obtained the same result as in the human challenge model, thus concluding that the effect of the importance of this efflux pump was strain dependent. As a whole the manuscript is easy to read and the data well presented. The major flaws of this manuscript which I would like to stress have already been discussed by the authors and unfortunately due to the use of the human model and the subsequent regulatory constraints cannot be experimentally address. The use of the strain FA1090 which is unable to upregulate the expression of the mtrCDE locus due to a spontaneous deletion in MtrA is considerably reducing the message of this manuscript.

We thank the reviewer for their positive feedback on our manuscript. We agree that FA1090’s reduced expression of the MtrCDE efflux pump relative to other gonoccocal strains can be seen as a limitation for its use in the study of the MtrCDE efflux pump. Perhaps a clearer explanation of the added value of our findings might alleviate some of the reviewer’s reservations about the generalizability and significance of our results with respect to the requirement of the Mtr efflux pump to the establishment of infection because it reveals important information about *N. gonorrhoeae* biology and vaccinology efforts related to an MtrCDE vaccine target.

First, though FA1090, the only human challenge strain currently approved for use in human challenge studies of gonorrhea by the US Food and Drug Administration IND authorization, does not have the ability to upregulate the expression of the pump, this characteristic is not uncommon among naturally occurring and currently circulating strains of *Neisseria gonorrhoeae*. A recent global genomic study of naturally occurring gonococci strains has shown that 7.5% of naturally occurring gonococci currently in circulation have an MtrCDE efflux pump with non-inducible expression through the same deletion as the one present in FA1090 and 3.8% of isolates have mutations in the *mtrCDE* operon that likely result in the loss of MtrCDE pump activity (Ma et al., 2021, Nature Communications, PMID 32807804). Because of this reason, we believe our results are directly relevant to at least 10% of naturally occurring gonococcal strains identifies to date globally. Second, global *N. gonorrhoeae* strains with mutations that downregulate or abrogate MtrCDE efflux pump activity were overrepresented in cervical *vs*. urethral isolates, suggesting that our results may be generalizable to human female genital infection, though this line of inquiry will not be able to be tested because of safety limitations of using the human model of infection in human females (i.e. risk of ascending infection). FA1090 was originally isolated from the endocervix of a female with probable disseminated gonococcal infection (please see lines please see lines 361-388 in our manuscript where we have amended our discussion to make this clearer).

Third, our findings have implications for development of a vaccine that targets MtrCDE. Combined, our data and those of the global genomic analyses of mutations in the *mtrCDE* operon and regulatory regions, demonstrate it is unlikely that a vaccine targeting MtrCDE will prevent infection acquisition, at least in the male urethra. It is possible that antibody against MtrCDE could still contribute to protection through targeting of *N. gonorrhoeae* for antibody mediated killing or by downregulating or inactivating MtrCDE efflux pump activity. Host immune mediated inactivation or downregulation of MtrCDE could render the bacteria more susceptible to antimicrobial substances, including antibiotics, and promote natural clearance of gonococci after infection was established. The idea of an MtrCDE-based vaccine as adjunctive therapy to antibiotics is supported by evidence that that loss of MtrCDE function in clinically-resistant gonococci that overexpress the pump increases their antibiotic susceptibility. The emergence of drug resistance and the dwindling arsenal of approved antibiotics that can effectively treat gonorrhea highlights the need for alternative gonorrhea control strategies (please see lines 390-401 in our manuscript where we have amended our discussion to make this clearer).

Finally, FA1090’s inability to upregulate the expression of the pump has enabled the identification of the colonization bottleneck in the human male urethra because wild-type FA1090’s decreased pump expression phenotype did not put it at a competitive advantage over the isogenic mutant with absent pump expression (lines 403-422); we believe the difference between lowly expressed MtrCDE *vs*. no expression of the MtrCDE pump was insufficient to alter fitness essentially leading to a situation where we conducted mixed inocula infections with two strains of comparable fitness, the gold standard of infection bottleneck investigations. This is the first time an infection bottleneck has been described for *N. gonorrhoeae* infection and to our knowledge for any bacterial sexually-transmitted infection. This is an intriguing novel aspect of *N. gonorrhoeae* biology that should be explored further because it has the potential to help identify new ways to control the infection by disabling gonococci from overcoming the infection bottleneck in the first place.

Reviewer #3: The authors present a comparative study that explores the behavior of N. gonorrhoeae in competitive index assays in men and laboratory mice. The influence on in vivo fitness of mtrD locus mutations in two gonococcal strain backgrounds was evaluated in mice; one strain background was evaluated in men. Valuable controls in men included mono-inoculation studies that helped determine that strain mixtures used in competitive index assays did not vary greatly in infectivity, inoculum size or pyuria. The bulk of the manuscript focused on competitive index assay data. Unexpectedly, mtrD mutations in the FA 1090 background did not impact fitness in the human challenge model of urethra infection. This differs from results in a murine model of vaginal infection where mtrD mutations impair FA19 strain fitness in vivo. This study builds on decades of previous work and is to be commended for attempting to merge human and murine infection models’ ability to inform knowledge of host-bacteria interactions that can help in design of vaccines and therapies.

We thank the reviewer for this summary and the positive feedback for the significance of our findings.

Competitive Index assay data would foster additional confidence if it was presented in a longitudinal format for the human and mouse studies. It was not clear why the authors only reported competitive index data for “final positive culture days” in humans and mice. Longitudinal data is presented in Figure 2B for the male volunteers but it would be nice to see (where possible) the competitive index data presented longitudinally for days post-inoculation using the format of data in Figure 1. This is a typical way to present competitive index data in at least one reference cited by the authors (such as reference 8, PMID: 18761689).

We thank the reviewer for this useful suggestion. We agree that Figure 1 could benefit from the inclusion of longitudinal competitive indices for both humans and mice. These have now been included for each set of competitive infection human and mouse experiments in the updated Figure 1, with relevant edits and updates in the results section and figure caption as well.

To clarify, the human competitive indices shown in the original Figure 1 reflect the proportion of strains identified on treatment day, which is the day when infection became symptomatic through the detection of clinical urethritis, i.e. when urethral discharge was observed by the study physician. This triggers an antibiotic treatment, making this the last day of post-inoculation follow-up at which gonococci were cultured before the participant exited the study. A clinical NAAT test of cure without bacterial culture was administered 2-3days after treatment day. The assumption is that by treatment day when the study endpoint was reached, namely clinical urethritis, the “winning” strain is the strain that successfully established a site of infection by outcompeting the “losing” or less fit strain due to its fitness competitive advantage. Thus, the competitive indices from the treatment study visit are the most biologically and clinically relevant at describing the results of the competitive infections (lines 217-221 in the manuscript). With respect to the mice, the CIs in the original Figure 1 reflect the strain composition at the last day of positive cultures. Female mice typically clear the colonizing bacteria within 5-7 days of challenge. In our study, we collected mouse vaginal swabs on day 1 post-inoculation and every other day (day 3, 5, and 7). All mice cleared the infection by day 7 and their last day of positive cultures was day 5. The assumption here is that the bacteria on the last day of bacterial cultures are the ones that were the strongest colonizers. For this reason, we chose this timepoint as the most relevant timepoint to assess the results of the mouse competitive infections. This is an established approach in the gonorrhea field for studies that employed the strategy of competitive infections and the mouse model of infection. We have included a better explanation of this in our manuscript (line 690-694).

The authors acknowledge that their data could be influenced by multiple factors including model systems, strain backgrounds and how a mutant strain was generated. I was not fully convinced that other variables also contributed to some of the differences noted. A more compelling presentation of bottleneck effects in the FA 1090 murine model would increase my enthusiasm for the study.

We appreciate the point that the reviewer has raised; however, our mouse data show that compared to human males, both strains are more likely to be recovered together. This suggests the findings of a bottleneck are not generalizable between the two species and that either the bottleneck does not exist in the murine model of infection or the bottleneck is wider/less restrictive in ways that are not evidenced in experiments using only two strains of close to equal fitness. It is worth noting that our study demonstrates an additional limitation in the murine model of *N. gonorrhoeae* infection that does not align with actual human infection (at least human male urethral infection), which is unsurprising given humans are the true and exclusive natural host of this pathogen. We may have failed to adequately communicate this point and we hope that our revisions of our manuscript at lines 454-464 better explain now the generalizability of the bottleneck observation to mice and the fact that we cannot generalize our findings to female humans due to limitations in the safety of the model.

Part II – Major Issues: Key Experiments Required for Acceptance
 Please use this section to detail the key new experiments or modifications of existing experiments that should be absolutely required to validate study conclusions.

Generally, there should be no more than 3 such required experiments or major modifications for a "Major Revision" recommendation. If more than 3 experiments are necessary to validate the study conclusions, then you are encouraged to recommend "Reject".

Reviewer #1: None

Reviewer #2: As mentioned by the authors a bottleneck is identified using two derivatives of the same strain with identical fitness. In this manuscript the authors aimed at assessing whether there is a different of fitness and concluded with a strain unable to fully expressed the WT phenotype that there is no competitive advantage of the locus under study but on the other hand identified a bottleneck. From the experimental design I have to admit I had to take some time to convince myself that this conclusion was correct. However, from the data with FA1090 in mice, this bottleneck does not seem completely as obvious in the mice model. It would be very nice if the authors could at least clearly identify this bottleneck in the mice model using two strains with the very same fitness.

We thank the reviewer for this important comment, which is similar to a comment from one of the other reviewers (see our response directly above).

Our mouse data show that compared to males, both strains are more likely to be recovered together, raising questions regarding the generalizability of the findings between the two species. This result may indicate that either the bottleneck does not exist in the murine model of infection or the bottleneck is wider/less restrictive in ways that are not evidenced in experiments using only two strains of close to equal fitness. Second, it is worth noting that our study demonstrates an additional limitation in the murine model of *N. gonorrhoeae* infection that does not align with actual human infection, which is unsurprising given humans are the true and exclusive natural host of this pathogen. We may have failed to adequately communicate this point and we hope that our revisions of our manuscript at lines 454-464 better explain the generalizability of the bottleneck observation to mice and human females (such as the fact that we cannot generalize our findings to female humans due to limitations in the safety of the model).

Reviewer #3:

Questions and comments.
 1. Regarding the inoculum descriptions such as those presented in Supplementary tables 2 and 3:
 a. Was each group inoculated with the same lot of inoculum? It was not clear if the each group received the same inoculum lot from the same frozen stock.

Yes, each group of human participants was inoculated with bacterial suspensions prepared from the overnight growth of bacteria plated from a thawed single use frozen vial of a working bank that contained the same lot of bacterial stocks.  For any given group, a single preparation of inoculum material was used within 15 minutes of preparation for all individuals inoculated in that group.

We have included clarifications in our manuscript regarding the bacterial lots/working banks at lines 511-538, and 605-607.

b. Was the age of the frozen inoculum or inocula used for the male volunteer studies between April and November of 2018 quite variable? It was not clear if study participants or mice received inoculum or inocula of widely different ages (stored at -70C for widely different amounts of time).

It is important to note that inocula in our studies are not used frozen directly out of the freezer. The inoculum that is administered to humans or mice is actually prepared from bacteria freshly growing on GCB plates for 18-24 hours hours prior to preparation of the inoculum. The freshly prepared inoculum is used within 15 minutes of preparation. The frozen stock of bacteria plated for use in the inoculum preparation for each cohort of inoculees (whether humans or mice for the experiments described in this manuscript) comes from a working bank containing single use vials created from a single lot of bacteria for each inoculum type (FA1090, FA1090Δ*mtrD*, or FA1090/ FA1090Δ*mtrD* mixed). The working bank of single use vials for each inoculum type was generated prior to the initiation of the study using that inoculum type and is extensively characterized and described in a Chemistry Manufacturing and Controls (CMC) document that details the initial characterization of each working bank lot, as well as required documentation of storage temperature, and regular stability testing. The CMC document for a lot of *Neisseria gonorrhoeae* strain FA1090A26 (where A26 identifies the lot of FA1090) was submitted with the initial FDA Investigational New Drug application and the CMC document was updated for each strain and strain mixture used in the study. The preparation of inocula has been extensively described in our prior publications. We have modified our description of the preparation of inoculum in the current manuscript to make the description of the production procedure clearer at lines 570-582.

Further information for the reviewer regarding the frozen single use vials of *N. gonorrhoeae* that were plated to provide the actively growing bacteria used in inoculum preparation include:

- the stability of the working bank lot was tested by enumerating cfu/mL from a single use vial every 18 months and results submitted to the FDA and no declines in stability were found during the study period.
- While the age of frozen bacterial product used to prepare the inoculum for the participants in this study was stored for variable amount of time, those frozen stocks had documented stability and the inoculum introduced to each human (or mouse) was prepared from actively growing bacteria that had been plated from a frozen stock for a uniform growth period.
- The studies with single infections took place first (April 2017 until May 2018) and inoculations with mixed inocula took place over 8 months in 4 separate cohorts (May 2018-November 2018).

c. Was the % of each strain in the strain mixtures quantified every time a stock was thawed prior to an inoculation?

Yes, the % of each strain in the strain mixtures was quantified for each preparation of inocula with one preparation of inoculum being generated for each group/cohort. These data are provided in Supplementary Table 2. The Competitive Indices reported for each participant was calculated with the exact proportion of strains identified in the inoculum of the group/cohort they were a part of. We have included a more detailed explanation of this at lines 670-672.

2. Were any of the same lots of inoculum used for inoculation of men used in mice or were they completely different lots? If different lots were used were they prepared in the same lab from the same founder stocks? This type of information in the methods section would be appreciated.

Yes, for strains that were used to inoculate both men and mice, the same lot of bacteria were used to make the inocula. Human challenge studies were conducted at the University of North Carolina at Chapel Hill, North Carolina. The mouse challenge studies were conducted in the laboratory of Dr. Ann Jerse at Uniformed Services University, Bethesda, Maryland. While men and mice were not inoculated with the same freshly made inoculum suspension, the same lots of bacteria and inoculum preparation procedures were used.

We have now included this explanation at 512-539 and 607-608.

3. Was the in vitro fitness of the strains created for this study analyzed? If not, should this be mentioned in the manuscript? If yes, could the authors describe the in vitro characterization?

The two strains (FA1090 and FA1090Δ*mtrD*) had equal growth rates when compared side by side in liquid cultures. We have now included this information in the methods of our manuscript in the strain characterization section.

In addition, we have conducted a liquid culture competitive growth experiment of the same stock of FA1090 and FA1090Δ*mtrD* mixture as used in the human and mouse challenge studies. This experiment was conducted in triplicate (i.e. using three individual vials of bacteria from the same working bank lot of FA1090 and FA1090Δ*mtrD* mixtures as used in human and mouse competitive infections). The procedure was as follows: three vials of FA1090 and FA1090Δ*mtrD* mixtures were thawed and bacteria were sub-cultured onto five individual GCB agar plates for each vial (15 plates total). The next day, three inoculum suspensions were made using the same procedures as for *in vivo* challenge studies. The three replicate bacterial suspensions in 6mL sterile PBS with an OD550 of ~0.3 were placed in liquid culture (34mL of GCB with supplements). Every 2 hours up to 8 hours, 4 mL aliquots of liquid culture were removed and centrifuged to obtain culture sediments. DNA from culture sediments was extracted. The DNA of the 0h, 4h and 8h timepoints were whole genome sequenced on the Illumina platform (2x150bp). Paired quality filtered reads were iteratively mapped to two types of FA1090 reference sequences with 5 iterations: one with an intact wild-type *mtrD* gene (wild-type reference sequence) and one with deleted *mtrD* gene (mutant reference sequence). Both reference sequence regions included 75bp 5’ and 3’ wild-type flanking sequences of the *mtrD* gene strains to allow for the mapping of the gene+flanks overlap (wild-type calls) or for the mapping of the deletion+flanks (mutant calls). The results of the whole genome sequencing are shown below. Some of the final sequencing data (8h) are pending and are to be released by the vendor the week beginning Sept 3. The available sequencing data show that there is no *in vitro* competitive fitness difference between the two strains.

| **Vial ID** | **Number of mutant CFU in inoculum** | **Number of wild-type CFU in inoculum** | **Mutant CFU inoculum / wild-type CFU inoculum** | **Number of FA1090∆*mtrD* mutant CFU after 4h of in vitro growth** | **Number of FA1090 wild-type CFU recovered from urine** | **Mutant CFU output from urine / wild-type CFU output after 4h of in vitro growth** | **Competitive Index (CI) (Column D/Column G)** | **Log10 (CI)** |
| --- | --- | --- | --- | --- | --- | --- | --- | --- |
| vial 1 | 2514 | 2340 | 0.93 | 2648 | 2418 | 1.10 | 0.85 | -0.07 |
| vial 2 | 2456 | 2654 | 1.08 | 2648 | 2511 | 1.05 | 1.02 | 0.01 |
| vial 3 | 2267 | 2413 | 1.06 | 1864 | 2012 | 0.93 | 1.15 | 0.06 |

4. On lines 227-228 it is mentioned that CFUs were measured up to five days after inoculation. Why is the competitive index data not reported for the earlier time points? The methods section reports that swabs were collected every other day but only data from the final sampling is reported. It would build confidence in the study if similar trends in the competitive index data were found to occur at multiple time points post-inoculation.

We agree that longitudinal data should be included. Daily CIs for humans and mice are now included in Figure 1. The swabs collected every other day were collected from mice not humans, who were sampled daily, with first void urine for culture as the clinical specimen.

5. Why were there not supplementary tables provided to show the strain percentages in strain mixture inocula used for FA19 studies? It seems like that would help the authors compare if PCR and culture-based measurements of competitive index yield similar data.

This information is now included in Supplementary Tables 4 and 6.

6. For figure 2, could the authors add a panel C to present similar data from the FA 1090 infected mice over time? It seems like the authors are contending the FA 1090 data supports the existence of a bottle neck in man and mice.

The longitudinal mouse data have now been included in Figure 1B, C, and D and in the supplement.

With respect to our conclusions about the bottleneck we have a few clarifications. We hope that we have made these points clearer in the discussion of the manuscript as well at lines 454-4694,, essentially that a bottleneck exists in the male urethral infection (the natural host of *N. gonorrhoeae* infection) and that there is not evidence for that bottleneck in the murine cervicovaginal infection model.

First, we do believe our data demonstrate the existence of a bottleneck in the initial stages of human colonization/infection. The murine data do not overlap with the human data 100%, but we wouldn’t expect them to for two main reasons: (1) mice are not this pathogen’s natural host and no doubt different pressures (or lack thereof) exist in this setting to influence the early course of infection. This is likely why mice also end up clearing the infection within 5-7 days. And (2) we are comparing the human male urethra to the mouse vagina, so in addition to species differences we’re also contending with biological sex differences. Is a gonococcal bottleneck also present in the human female lower genital tract? We cannot answer this with any level of certainty and this is a limitation of our observation because risk of an ascending infection means we cannot and will not use the human model of gonorrhea in human females. We hope our revisions of our manuscript to better note this point and the limitations of the observation (such as the fact that we cannot generalize our findings to female humans due to limitations in the safety of the model).

7. I feel the 60% mutant/40% wild type pie chart in Figure 2B is slightly misleading. Would the authors consider adding pie charts for the inoculum each participant received on the left side of the panel (using the numbers from supplementary table 2)? This would help readers identify which participants received the same or similar inoculum. It might also be worthwhile to add the group number in parentheses behind each participant number so readers can correlate participants with information provided in supplementary table 2.

These are great ideas and Figure 2 has been revised with these changes.

8. The participants’ pie chart data in Fig. 2B appears to have been organized in a manner that resembles option 4 in Figure 2A. It is not organized according to group as in the supplementary table 2. I found Figure 2A a bit confusing because for options 2 and 3 only “Wild-type advantaged” was depicted. It seems like those options could be divided into parts A and B and you could depict both “Wild-type advantaged” and “mutant advantaged”.

We have now revised this figure to include these suggestions.

9. Lines 414-416. My reading of your manuscript led me to conclude you think there is a bottle neck for FA 1090 in the murine model too. Why can’t you add your mouse data as a panel C to Figure 2 to show how it compares to the human data?

We appreciate the point that the reviewer has raised. As in response to another reviewer comment, we’d like to clarify that we do believe our data demonstrate the existence of a bottleneck in the initial stages of human infection but it remains this does not appear to be the case in the mouse. As noted above in several places, perhaps we did not explain this with sufficient clarity and we have now made revisions to our discussion to state:

“It is a limitation of our study that we have not investigated the basis of the bottleneck using the murine model and future mouse studies could potentially explore this question and advance these initial findings. However, it is worth noting that our study shows that even under controlled conditions and with the use of extensively characterized and defined strains the mouse model of infection does not fully align with human infection. Even if a bottleneck is present in the mouse vagina, it will likely not replicate the characteristics of the bottleneck in the human male urethra and this will ultimately limit how generalizable those findings will be to human infection, the exclusive natural hosts of this pathogen. These differences are likely why in the established murine model of *N. gonorrhoeae* infection, mice clear the infection within 5-7 days. On the other hand, a bottleneck in the mouse that restricts *N. gonorrhoeae* colonization may be more generalizable to studies of infection bottleneck during human females genital infection, but due to limitations noted above in the safety of the gonorrhea challenge model for human females, it is not possible to pursue this line of inquiry.”

We hope our revisions of our manuscript to better note this point and the limitations of the observation (such as the fact that we cannot generalize our findings to female humans due to limitations in the safety of the model). We will be unable to provide additional experimental evidence requested to demonstrate the presence of a bottleneck in mice. Again, we emphasize that humans are the true and accepted exclusive natural host for this pathogen.

10. Is one way to interpret the data in Figure 2B that the mtrD mutants are capable of causing longer periods of asymptomatic urethritis? It seems like participants dominated by wild-type FA 1090 exited the study earlier (I assume - due to detection of urethritis and treatment). While fitness for both strains was not significantly different it seems like the two strains differ in their asymptomatic persistence and timing of urethritis symptoms.

## We thank the reviewer for this observation. We have noted this difference as well. The time to treatment of men whose infection course was dominated by the mutant strain was indeed longer than for men whose infection was dominated by the wild-type strain (4 days to treatment for mutant-dominated infections vs. 3 days to treatment for infections dominated by the wild-type strain, p=0.03, Table 3). This phenomenon was not replicated during single strain experimental infections (p=0.50, Table 2). When time to treatment was compared among all men from both single strain and competitive infection inoculations, no difference was observed between infections composed of or dominated by wild-type or mutant strain on treatment day (p=0.18). For men whose infection was initiated with the mixture of strains and was dominated by the mutant strain on treatment day, we generally did not observe a period of competition followed by emergence of the mutant as the dominant strain. We cannot think of a biological reason why strain differences would lead to this scenario. To formally answer this question would require a whole new clinical trial of competitive infections with these two strains, which is beyond the scope our work here.

11. Line 398. The references cited for single-cell bottlenecks are all quite dated. Tn-seq has been very helpful in elucidating bottlenecks for infectious bacteria in animal models. Many human bacterial pathogens have much larger bottle necks than single cell bottlenecks. I don’t find the bottleneck discussion very compelling and think it needs updated with more current examples from the literature.

We thank the reviewer for the careful examination of our citations. We believe the references pasted below are the ones the reviewer’s is raising concerns about. While citations 56-59 refer to studies that were published in the 1990s and 2000s, they were pivotal studies conducted by our group with the human challenge model that monitored the antigenic variation of key virulence factors during experimental infection. These findings were the genesis of the gonococcal bottleneck hypothesis. Not citing these would be a mistake. As for references 60-62, these studies (citation 60) cite the original article that documented for the first time the presence of a tight bottleneck in *Neisseria meningitidis*, the only other pathogenic species in the genus, and two others (61 and 62) discuss why the loci involved in describing the meningococcal bottleneck are also components of the gonococcus. We believe these are relevant to the current body of work, irrespective of the year they were published. Nevertheless, the point about the evolution of bacterial bottleneck studies to include newer, higher throughput technologies is valid, though not particularly relevant to our study utilizing the human model of infection. For example, Tn-seq (i.e transposon insertion sequencing), a technology that the reviewer has brought up, has been indeed used to study essential genes and shed light on bacterial bottlenecks under certain circumstances, mainly in animal models, including one that investigated essential genes of a murine neisserial commensal. This murine neisserial commensal study of oropharyngeal and gut colonization did not find evidence for a bottleneck at these anatomical sites after inoculation with a transposon library covering the genome, though limitations were noted. We have included in our discussion at lines 417-422 a commentary of such analyses to the study of bacterial colonization bottlenecks with animal models.

While Tn-seq and other high-throughput mutagenesis and screening approaches are a novel more powerful way to do conduct these analyses, its utility in studying bottlenecks in *in vivo* infections is limited by noisy data and by difficulties in tracking mutants over time, especially if the complexity of the pool is as large as typical Tn-seq experiments in bacteria tend to be. These limitations of noise and stochastic events were cited as limitations by the murine commensal study. Infection studies with lower complexity pools, and especially with mutants containing clean deletions which are the only option in human challenge studies, like the one used in our work, may be lower throughout, but better suited in demonstrating the presence of a colonization bottleneck. Finally, as attractive and compelling as it would be to study the competitive advantage of multiple gonococcal strains in experimental human infections, the use of Tn-seq and similar technologies is unclear from a regulatory and safety point of view, because an IND for a GC product with hundreds or thousands of variants will likely not be accepted by FDA (our human challenge studies are conducted with FDA oversight with an IND).

12. Is it worthwhile to add supplementary graphs showing longitudinal CFUs/ml of urine for the human participants and CFUs/vaginal swab for each mouse. It might be another way of illustrating CFU burden differences between the two models.

We have now included supplementary tables of mouse longitudinal data.

13. Figure 1 could be improved if the symbols could be annotated with the day post-inoculation the data came from. Could a superscript be added to each symbol for the day post-inoculation the data came from? Alternatively, could different colored borders be added to each symbol and a legend added indicating the day post-inoculation the competitive index data came from.

These are great ideas and Figure 1 has been revised to include these changes.

Part III – Minor Issues: Editorial and Data Presentation Modifications
 Please use this section for editorial suggestions as well as relatively minor modifications of existing data that would enhance clarity.

Reviewer #1: 1. General. It is usual practice to state the age (range, mean + SD) and ethnicity of the human volunteers who participated in the infection experiments. Please add that data to the text or Tables.

2. Line 147, correct “condidates”

Done.

3. Line 152, insert a comma before “and”

Done

4. Line 161, complete the sentence “demonstrate a gonococcal infection bottleneck for the first.”

Done.

5. Line172, it is unclear whether FA1090 A26 (which lacks MtrA expression) is considered to be the “wild type” strain or is a derivative of FA1090. Please clarify. If you choose to use the A26 designation, please use it throughout (for example, FA1090 is used in Table 2, not FA1090 A26). I happen to know that the A26 designation refers to a stock of FA1090 that is used for human challenges due to its defined pilation and opa states, but the general reader will not know this, and we don’t find that out until line 447 in the methods.

We thank the reviewer for picking up on this very important point. We have now included in the methods of the manuscript an explanation of the A26 designation as the working bank of wild-type FA1090 that was generated and lot released for use in human challenge studies (lines 510-543)

6. Line 194-195 , change “and thus processed urines were not the first void of the day” to “of providing first voided urine.”

Fixed.

7. Line 232. Please address discrepancies between text, Figure 1B and Supplementary Table In text, it is stated “we observed five outcomes that favored the wild type FA1090 and six outcomes that favored FA1090ΔmtrD, but the supplementary table indicates there were only 10 evaluable mice, not 11, with 5 outcomes that favored the wild type and 5 outcomes that favored the mutant. Figure 1B also has 11 outcomes.

Fixed.

8. Figure 1 text states it is using horizonal bars for medians of each group, but it also appears to be using the same horizontal bars for the CIs (not stated in the legend); use of the same symbol for different purposes is confusing.

We apologize for the confusion; this has now been clarified in the figure caption.

9. Fig 2B. Please move the pie chart showing the inoculum to the left of the Figure panel (currently it is within the panel and aligns with volunteer 313)

Fixed.

10. Line 290 is this a polar mutant?

Yes.

11. The first paragraph of the discussion is nearly 50 lines long. Please consider a paragraph break after you finish discussing the murine data in line 311 and have a separate paragraph about the human data.

Done.

12. Line 384, 385, 415, 425 fix “lackthereof” , “variants variants” "porentially", “sexually-transmitetd”; please re-check for typos throughout

Fixed

13. Lines 418-23 the statement “that by showing gonococci can initiate productive infection in the human male urethra even when the pump is not functional, we have generated data that can inform future gonorrhea vaccine and drug development efforts that target the function of the Mtr efflux pump” is vague. Please expand the discussion to state whether the Mtr pump remains a reasonable drug or vaccine target, given all the human data you present here.

We thank the reviewer for bringing up this interesting point. Based on the data of our current study and those of the global genomic analyses, which show that naturally occurring strains that express the pump at low levels (like FA1090) or have loss of function mutations are common in nature and currently circulating around the world, it is unlikely that a vaccine targeting MtrCDE will provide sterilizing immunity or be broadly effective. However, it is not out of the realm of possibility that under certain circumstances antibody against MtrCDE may be protective; for example, in women it could reduce or block export of host-derived antimicrobials and render gonococci more susceptible to clearance by these host compounds. In this respect, the results with the human male challenge may not extend to naturally infected women. In addition, it is possible that a vaccine that targets MtrCDE, though not protective against infection, could modulate antibiotic susceptibility in the host and be used as an adjunctive therapeutic vaccine to improve treatment outcomes with currently available antibiotics. We hope our revision of the discussion at lines 390-401 addresses the previous gap.

It would also be better if the last paragraph of the discussion were split into 2 parts, one focused on the pump and the last focused on the bottleneck, which deserves to stand on its own, as that may be the most important finding here.

We agree and we have now revised the discussion to include separate sections for the two pieces of information we generated in our study.

Reviewer #2: The last part of the discussion is not really necessary.

Agree and we have edited the manuscript to cut down on this redundant part of the discussion.

Reviewer #3: Line 147. Correct spelling of “condidates”.

Fixed.

Line 218. “two models” – I assume this refers to the human challenge model and the BALB/c mouse model. It is easy to get lost and might help readers like myself to edit “two models” to “human and murine models showed similar results”.

We thank the reviewer for pointing this out. We have revised this section to be clearer and it now reads: This result recapitulates the results of the human competitive infections, indicating that FA1090Δ*mtrD* does not have a fitness defect during *in vivo* infection of men and mice. The lack of a fitness defect was also noted when FA1090 and FA1090Δ*mtrD* were grown competitively *in vitro* in liquid cultures. One difference between the results of the human and mouse competitive infections with when FA1090 and FA1090Δ*mtrD* was that more mice than men had mixed strain recovery on the final positive culture day (mice: 6 /11, 54.5%; men: 1/10, 10.0%, *p=*0.045 one-sided Fisher’s exact).

Line 424. Correct spelling error – “transmitetd”.

Fixed.
Line 594. The timing of the human infections is listed but not the murine experiments.

Fixed.

Line 689. It would be helpful to adjust the legend or panel 1A so it was clear that the numbers in panel 1A refer to “infection outcomes”.

Thank you for this suggestion.

Lines 625, 643. “wbc” not defined.

Fixed

“Tx” not defined in supplementary tables.

Fixed

Fig. 1B “FA 1090” is missing from the upper text on the right side of the graph.

Fixed.

I wish the authors could add something to their discussion about “transmission.

We thank the reviewer for this important suggestion. We have revised our discussion at lines 474-487 to include a commentary on the implications of the bottleneck finding on transmission and it states:

“Reductions in the genetic diversity of pathogen populations can impact their transmission fitness. Our controlled human model of infection is not developed to study transmission, and it is currently unknown whether the bottleneck we observed during human experimental urethral infection impacts transmission of gonococci. It is worth noting that phase and antigenic variation have long been a defining characteristic of *N. gonorrhoeae* and the recovery of antigenically heterogenous gonococci from the urethra of experimentally infected males relative to the antigenic characteristics of inoculating gonococci ([52-55](bookmark://_ENREF_52)), as described above, speaks to the possibility that *N. gonorrhoeae* deploys phase and antigenic variation in response to the host bottleneck to ensure a diverse population is fit for onward transmission to a new site of infection. Future studies aimed to reveal which host factors drive bottleneck or which genes are essential to the survival of *N. gonorrhoeae* under bottleneck conditions will begin providing the information needed to explore whether the bottleneck can be exploited to thwart *N. gonorrhoeae’s* ability to establish an initial infection and transmit to the next site of infection.”
